# Supplementary material for: Seminoma and Embryonal Carcinoma Footprints Identified by Analysis of Integrated Genome-Wide Epigenetic and Expression Profiles of Germ Cell Cancer Cell Lines
Source: PLoS One. 2014 Jun 2;9(6):e98330. doi: 10.1371/journal.pone.0098330 (PMC4041891; doi:10.1371/journal.pone.0098330)
Supplement: File S1 — ZIP file containing DMRforPairs output for significant regions. Please start from the html files. (ZIP) [file pone.0098330.s008.zip › figures/31283.pdf]

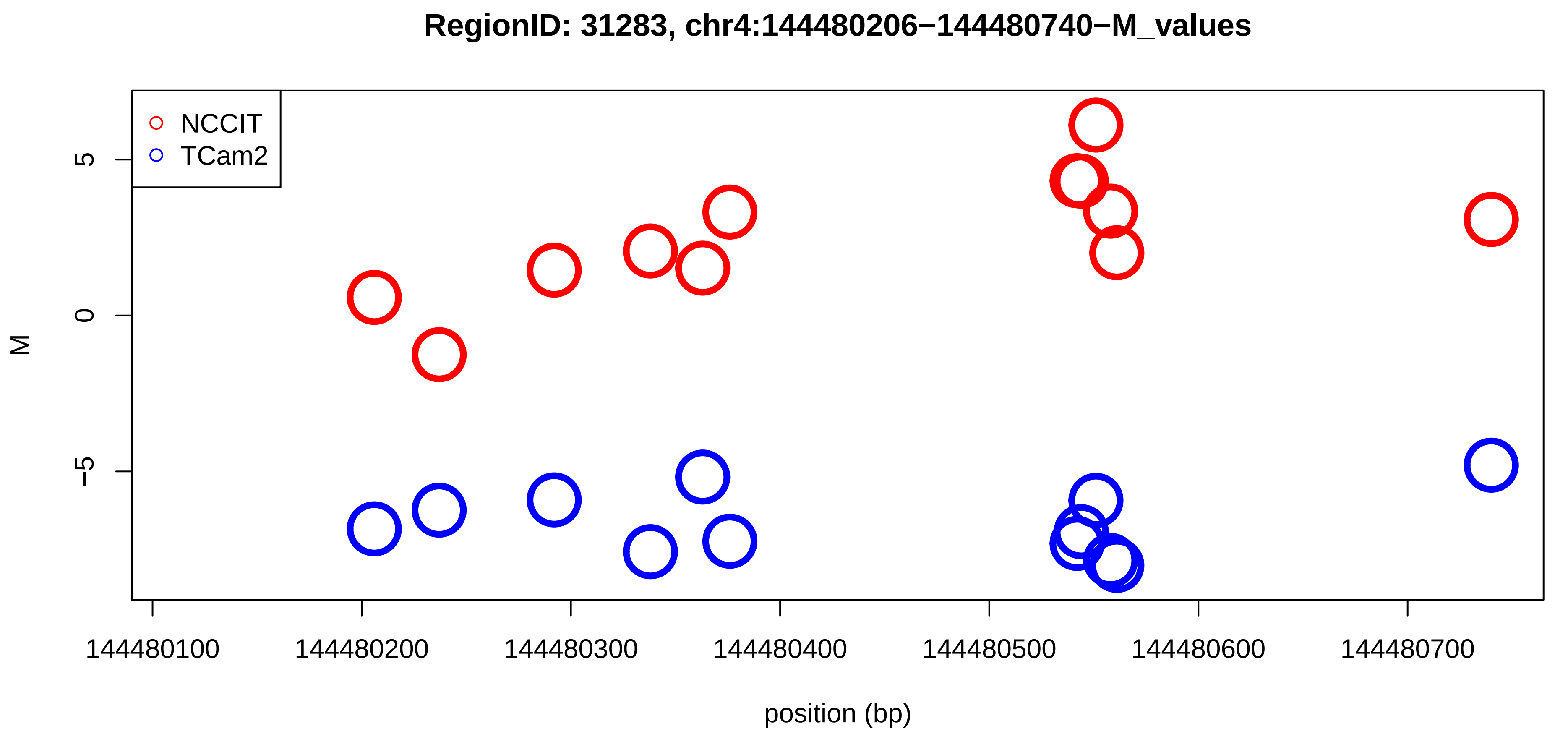
RegionID: 31283, chr4:144480206−144480740−M_values
NCCIT
TCam2
M
5
0
−5
144480100
144480200
144480300
144480400
144480500
144480600
144480700
position (bp)

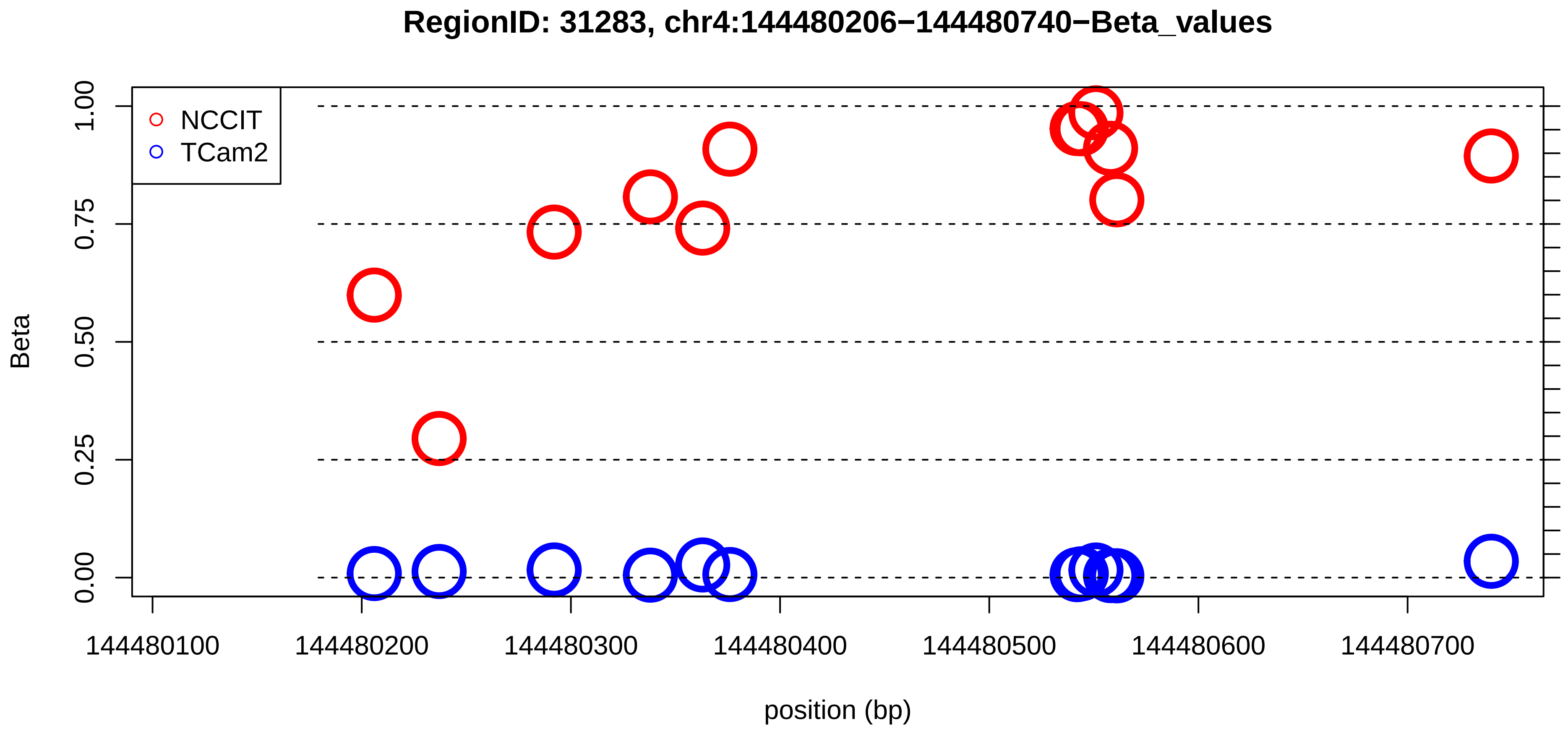
RegionID: 31283, chr4:144480206−144480740−Beta_values
NCCIT
TCam2
Beta
1.00
0.75
0.50
0.25
0.00
144480100
144480200
144480300
144480400
144480500
144480600
144480700
position (bp)

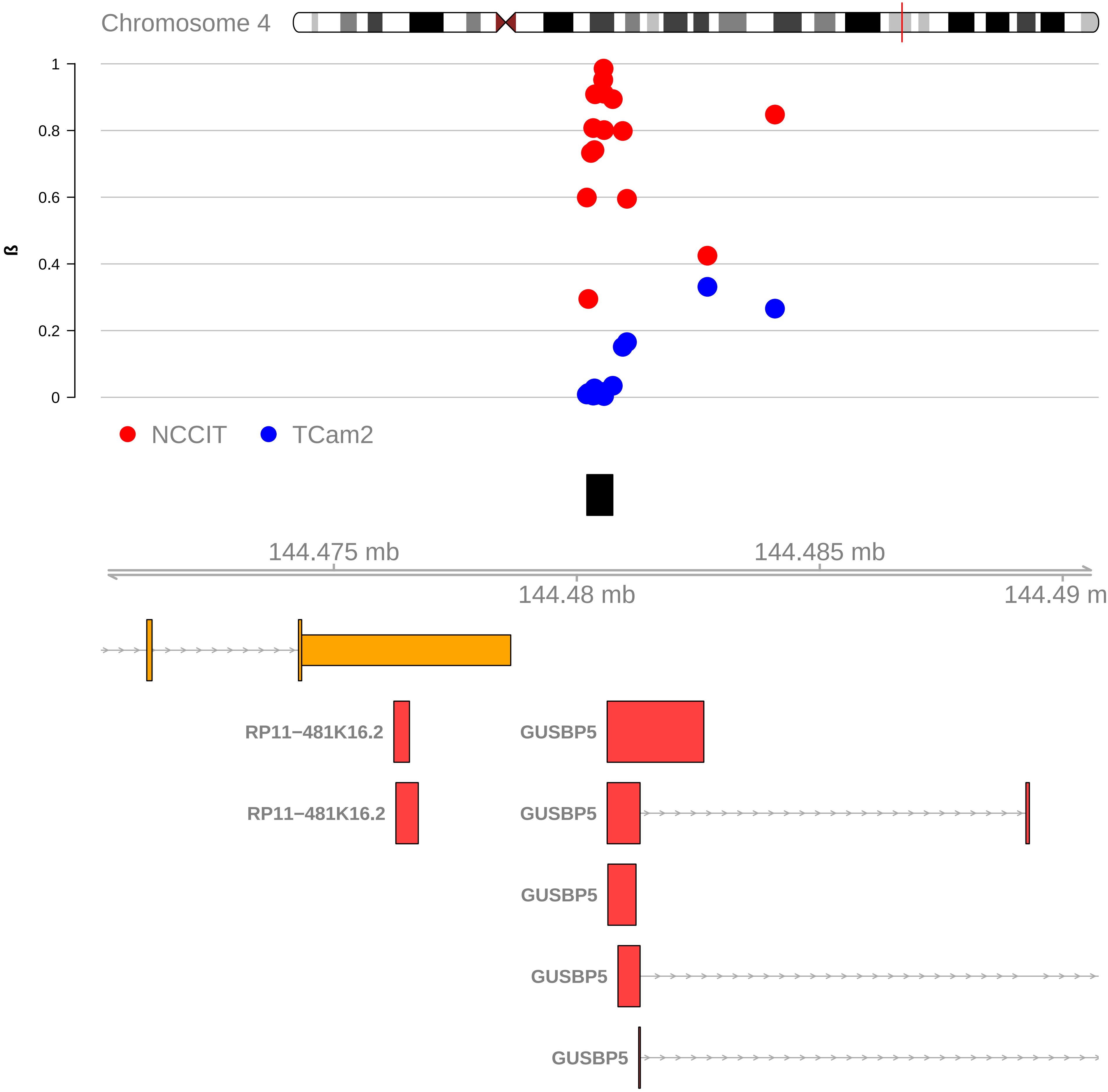

Chromosome 4
1
0.8
0.6
0.4
0.2
0
ß
NCCIT
TCam2
144.475 mb
144.485 mb
144.48 mb
144.49 m
RP11−481K16.2
GUSBP5
RP11−481K16.2
GUSBP5
GUSBP5
GUSBP5
GUSBP5
